# Exercise: The key to enhancing sleep quality and physical function in Parkinson’s disease? A systematic review and meta-analysis

**DOI:** 10.1371/journal.pone.0336381

**Published:** 2025-11-26

**Authors:** Zhiqin Li, Pingqing Hu

**Affiliations:** 1 China Wushu School, Beijing Sport University, Beijing, China; 2 Graduate School, Beijing Sport University, Beijing, China; University rehabilitation institute, SLOVENIA

## Abstract

Parkinson’s disease (PD) is a significant neurodegenerative disorder that affects millions of individuals worldwide and currently has limited effective treatment options. Exercise has been proposed as a non-pharmacological intervention to improve both motor and non-motor symptoms in PD. This study aims to systematically review and meta-analyze the impact of exercise interventions on sleep quality and physical functioning in PD patients. A comprehensive search of the literature up to December 15, 2023, identified randomized controlled trials that evaluated exercise interventions in PD patients. The primary outcomes were sleep quality, motor function, balance, gait performance, and quality of life. A total of 62 studies with 3,274 participants were included in the analysis. Exercise interventions led to significant improvements in sleep quality [SMD = −0.55, 95% CI (−0.91, −0.18), *p* = 0.003], motor capability [SMD = −0.47, 95% CI (−0.66, −0.28), *p* < 0.01], balance ability [SMD = 0.53, 95% CI (0.33, 0.74), *p* < 0.0001], gait performance [TUGT: SMD = −0.44, 95% CI (−0.60, −0.29), *p* = 0.0017; stride velocity: SMD = 0.38, 95% CI (0.15, 0.60), *p* = 0.001; step length: SMD = 0.32, 95% CI (0.10, 0.54), *p* = 0.004], and quality of life [SMD = −0.38, 95% CI (−0.73, −0.03), *p* = 0.04] (**p* *< 0.05). Exercise is an effective intervention for enhancing sleep quality and improving physical function in PD patients. These findings underscore the importance of incorporating exercise into the management strategies for PD.

## Introduction

Parkinson’s disease (PD) is a challenging public health issue that urgently needs to be addressed, as it is one of the leading causes of disability and mortality among the elderly. PD is associated with a variety of factors and is a complex neurodegenerative disorder that faces significant clinical diagnostic and therapeutic obstacles, including the inability to make accurate diagnoses in the early stages of the disease and difficulties in treatment during the later stages. The clinical manifestations of PD primarily include resting tremor, bradykinesia, muscle rigidity, and postural gait disorders, while symptoms such as depression, constipation, and sleep disturbances may also be present [1]. Rapid Eye Movement (REM) sleep behavior disorder, excessive daytime sleepiness, and insomnia are among the most common non-motor symptoms in patients with PD, with a prevalence rate ranging from 9% ~ 83%. Moreover, up to 52% of early-stage PD patients may experience sleep disturbances [2]. Sleep disorders can negatively impact nocturnal rest, motor function, and overall quality of life in PD patients [3]. According to data from the National Parkinson Foundation (NPF), there are approximately 10 million PD patients worldwide, with an incidence rate of about 4.5 ~ 19 per 100,000 persons [4]. China accounts for approximately one-third of the global PD population, with a PD prevalence rate of 1.7% in the population over 65 years old, and the number of patients is increasing at a rate of about 100,000 people per year. By 2030, the number of patients is expected to reach nearly 5 million [5]. Consequently, PD has emerged as the “third major killer” threatening the health of the elderly, following malignant tumors and cardiovascular and cerebrovascular diseases. The quality of life for patients is severely affected by this condition, imposing a significant burden on both their families and society. Despite advancements in understanding the pathogenesis of PD, an effective clinical treatment remains elusive. The challenges associated with drug development for PD have necessitated a reevaluation of prevention and treatment strategies for this complex disorder from novel perspectives.

Exercise is an important non-pharmacological intervention for PD. It offers the advantages of being efficient, economical, and accessible. According to the American College of Sports Medicine (ACSM), exercise is a planned, structured, and repetitive bodily activity aimed at maintaining or improving one or more components of physical health. Studies have shown that exercise has a significant neuroprotective effect, capable of delaying the onset and progression of PD [6]. Aquatic therapy is one type of exercise therapy that refers to conducting exercise or rehabilitation training and treatment in an aquatic environment. It can alleviate patient symptoms and improve motor function, and is now widely applied in neuromuscular rehabilitation [7]. Duchesne et al. [8] clinically demonstrated that aerobic exercise can improve motor function of PD patients; Li et al. [9] conducted a 24-week Tai Chi intervention for PD patients, with results indicating that exercise can enhance balance and gait performance while reducing the fall rate; Holmes et al. [10] adapted Tango training for PD patients, and their study showed that exercise can improve the daily living abilities and quality of life of PD patients; Kwok et al. [11] demonstrated that an 8-week resistance training program was effective in a group of 138 PD patients, with a significant clinical improvement in motor function. However, some studies have failed to confirm the effectiveness of exercise intervention for PD, such as Amano et al. [12], whose research showed that 16 weeks of Tai Chi exercise did not significantly affect the motor function of PD patients; Tillman et al. [13] found that resistance training did not improve the gait performance of patients. It is evident that the exercise intervention protocols vary, and the research outcomes are not consistent. Moreover, the existing meta-analyses are limited by small sample sizes, limited data, and potential publication bias, which may restrict the generalizability and reliability of the results [14,15]. Currently, there is a lack of persuasive analysis and evaluation regarding the effectiveness of exercise intervention for PD. In light of this, the purpose of this study is to conduct a meta-analysis to assess the impact of exercise on sleep quality and physical functioning in PD patients. This analysis will provide a theoretical and practical basis for exercise-based interventions in the prevention and treatment of PD.

## Methods

This meta-analysis was conducted in accordance with the Preferred Reporting Items for Systematic Reviews and Meta-analyses (PRISMA) reporting guideline [16].

### Literature search strategy

Five databases, including PubMed/MEDLINE, Embase, Cochrane Library, Web of Science and China National Knowledge Infrastructure (CNKI), were systematically searched up to December 15, 2023. The subject terms, combined with free-text keywords, were used in the retrieval strategy. The key terms were “Parkinson’s disease”, “exercise”, “aerobic exercise”, “yoga”, “Taichi”, “qigong”, “wuqinxi”, “resistance training”, “hydrotherapy”, “aquatic therapy”, “water-based exercise”. More relevant literature was collected. The study protocol was registered at PROSPERO (https://www.crd.york.ac.uk/prospero/) as CRD42024583992.

### Study selection

To be included in the systematic review and meta-analysis, studies had to fulfill the following inclusion criteria: the research subjects were adult patients diagnosed with Parkinson’s disease. Literature was excluded if it involved patients with non-primary Parkinson’s, if the full text was unavailable, if there was missing data, or if the publication was a conference abstract, dissertation, case study, or animal experiment. Additionally, studies involving patients with non-primary Parkinson’s, such as those with Parkinson’s Plus Syndromes or secondary Parkinson’s syndrome, were excluded. Intervention methods were not detailed; no outcome was available; pre- and post-measurement data could not be converted into mean and standard deviations, or duplicate literature. The study was designed as a randomized controlled trial (RCT), in which the experimental group received exercise training while the control group received routine care, with no intervention or land-based training. The language of the literature was Chinese or English. The outcome indicators of this study involved sleep quality (Parkinson’s Disease Sleep Scale, PDSS; Pittsburgh Sleep Quality Index, PSQI; Unified Parkinson’s Disease Rating Scale, UPDRS; Mini-Sleep Questionnaire, MSQ), motor ability (Unified Parkinson’s Disease Rating Scale-Motor Examination, UPDRS-III), balance ability (Berg Balance Scale, BBS), gait performance (“Timed Up To Go” Test, TUGT; stride velocity; step length), quality of life (Parkinson’s Disease Questionnaire-39, PDQ-39).

### Data extraction

Two reviewers independently screened the title and abstract of the studies and then performed full-text review and study selection based on the eligibility criteria. Data extracts included basic study information (first author, year of publication, sample size, age), intervention details (treatment duration, time, comparison interventions), and outcome indicators. The characteristics of the included studies are presented in Table 1.

**Table 1:** Basic features of included literature.

Authors	Year	Sample	Average age (Mean ± SD)		Hoehn-Yahr	Duration	Outcome	Intervention methods		Adverse events
			Intervention group	Control group				Intervention	Control	
Li [19]	2022	40	67.59 ± 3.95	70 ± 5.59	1-3	4week	①②	Wuqinxi	Stretching	No
Kong [20]	2022	92	66.1 ± 5.37	65.79 ± 4.12	1-3	3week	①②	Wuqinxi	Conventional therapy	Not mentioned
Wagner [21]	2022	230	64.1 ± 9.3	67.6 ± 9.3	1-2	9month	⑦	Aerobic exercise	Conventional therapy	Not mentioned
Shen [22]	2021	30	68.67 ± 4.33	66.93 ± 3.36	1-3	12week	①④	Wuqinxi	Stretching	No
Xiao [23]	2021	40	72.78 ± 2.63	72.58 ± 2.62	1-2.5	6month	②	Tai Chi	Conventional medical	Not mentioned
Wu [24]	2021	98	63.65 ± 6.02	66.59 ± 8.61	1-2	8week	⑧	Aerobic exercise	Regular lifestyle	Not mentioned
Nuan [25]	2020	26	64.08 ± 3.95	63.46 ± 4.33	1-2.5	16week	①②④	Tai Chi	Conventional medical	Not mentioned
Deng [26]	2020	60	54.5 ± 1.2	54.4 ± 1.3	1-2.5	12week	②	Tai Chi	Conventional medical	Not mentioned
Silva-Batista [27]	2020	32	64.6 ± 10.5	66.8 ± 8.9	3-4	12week	①③	Resistance training	Traditional motor rehabilitation	Acute sciatica
Zhu [28]	2020	41	68.53 ± 1.90	67.77 ± 1.72	1-3	12week	⑦	Tai Chi	Regular lifestyle	Not mentioned
Amara [29]	2020	55	65.33 ± 8.17	65.82 ± 5.19	2-3	16week	⑧	Resistance training	In‐person discussion and monthly phone calls	Not mentioned
Leal [30]	2019	54	65.2 ± 2.05	64.9 ± 2.32	1-3	6month	④	Resistance training	Pharmacological treatments	Not mentioned
Silva [31]	2019	25	63.12 ± 13.61	64.23 ± 13.45	1-4	10week	②④	Dual-task aquatic exercises	No intervention	Not mentioned
Liu [32]	2019	207	61.43 ± 10.12	60.15 ± 9.75	1-3	52week	①②	Baduanjin	Conventional therapy	Falls
Lima [33]	2019	33	66.2 ± 5.5	67.2 ± 5.2	1-3	20week	③④⑥	Resistance training	Conventional therapy	Not mentioned
Shen [34]	2019	85	64.87 ± 4.76	65.08 ± 4.91	1-4	18month	②	Resistance training	No intervention	Somnolence; nausea
Clerici [35]	2019	52	67 ± 8	67 ± 11	2-3	4week	①②④	MIRT+ Aquatic Training	MIRT+ Land Training	Urinary tract infection; water-choke discomfort
Coe [36]	2018	65	67 ± 7.12	67 ± 5.88	NR	6month	⑪	Aerobic exercise	Handwriting	Not mentioned
Elyazed [37]	2018	30	66.13 ± 5.66	65.27 ± 4.96	NR	12week	⑩	Aerobic exercise	Physical therapy	Not mentioned
Yang [38]	2018	36	58.95 ± 4.66	58.01 ± 4.62	NR	8week	②④	Resistance training	Rehabilitation training	Not mentioned
Ma [39]	2018	100	/	/	NR	1month	⑤⑥	Aerobic exercise	pharmacological treatments	Not mentioned
Wu [40]	2018	52	62.4 ± 5.37	64.66 ± 5.47	1-3	16week	③	Aerobic exercise	Pharmacological treatments	Not mentioned
Diaz [41]	2018	32	61.08 ± 4.41	60.48 ± 5.46	1-2	4week	①③④	Aerobic exercise	Usual care	back pain; falls/injuries; illness
Cheung [42]	2018	20	63.5 ± 8.5	65.8 ± 6.6	1-3	12week	⑦	Yoga	Wait-list	Not mentioned
Guan [43]	2018	80	69.46 ± 5.45	68.61 ± 6.22	/	24week	②④⑤⑥	Tai Chi	Usual care	Not mentioned
Ferreira [44]	2018	35	64.1 ± 7.0	67.6 ± 8.9	1-3	24week	①③	Resistance training	Standard pharmacological treatment	Not mentioned
Santos [45]	2017	28	73.38 ± 8.81	73.80 ± 7.05	1-2	8week	③⑤⑥	Resistance training	Routine activities	Not mentioned
Palamara [46]	2017	34	69.46 ± 5.45	68.61 ± 6.22	/	4week	①②④	MIRT+ Aquatic Training	MIRT+ Land Training	Not mentioned
Perez-de la [47]	2017	30	66.8 ± 5.26	67.53 ± 9.89	1-3	10week	②④	Aquatic therapy	Land-based training	No
Silva-Batista [48]	2017	22	64.6 ± 9.7	64.4 ± 9.1	2-3	12week	⑧	Resistance training	Nonexercising	No
Volpe a [49]	2017	24	/	/	1-3	3week	①②③④	Underwater	Land-based training	Not mentioned
Volpe b [50]	2017	30	70.6 ± 7.8	70 ± 7.8	1-3	8week	①②③④	Underwater	Land-based training	Orthostatic Hypotens
Zhu [51]	2017	16	63.5 ± 6.78	61.5 ± 5.63	2.5-3	12week	①③	Tai Chi	Stretching	Not mentioned
Memarian [52]	2017	24	/	/	1-3	8week	⑧	Yoga	Standard medical treatment	Not mentioned
Lu [53]	2017	16	67.75 ± 6.84	68.20 ± 7.32	1-2	8week	①②	Tai Chi	Conventional therapy	Not mentioned
Guan [54]	2017	80	69.46 ± 5.45	68.61 ± 6.22	1-2.5	24week	②	Tai Chi	Conventional therapy	Not mentioned
Carroll [55]	2017	18	/	/	1-3	6week	①③	Aquatic therapy	Conventional therapy	No
Altmann [56]	2016	29	62.8 ± 8.6	67.8 ± 9.8	1-3	16week	⑧	Aerobic exercise	Normal activities	Not mentioned
Silva-Batista [57]	2016	26	64.1 ± 9.1	64.2 ± 8.3	2-3	12wk	①③④	Resistance training	No intervention	Not mentioned
Ni [58]	2016	27	71.6 ± 6.6	71.2 ± 6.5	1-3	12wk	②④⑥	Yoga	Nonexercise	Not mentioned
Ji [59]	2016	38	56.06 ± 11.16	59.13 ± 11.22	1-3	3month	①②	Tai Chi	Conventional therapy	Not mentioned
Xiao [60]	2016	96	68.17 ± 2.27	66.52 ± 2.13	1-3	6month	⑦	Baduanjin	Standard therapy	Not mentioned
Guan [61]	2016	62	70.23 ± 4.24	69.71 ± 4.13	1-3	12week	①②	Tai Chi	Rehabilitation exercise	Not mentioned
Frazzitta [62]	2015	138	67.6 ± 7.2	71.8 ± 7.2	2-3	4week	⑦	Aerobic exercise	Routine activities	Not mentioned
Shen [63]	2015	45	63.3 ± 8.0	65.3 ± 8.5	2-3	3month	⑤⑥	Technology-assisted balance and gait training	Strengthening exercises	Acute bronchitis
Zhang [64]	2015	40	66 ± 11.80	64.35 ± 10.53	1-4	12week	①②④⑤⑥	Tai chi	Multimodal exercise	Acute bronchitis
Romenets [65]	2015	33	63.2 ± 9.9	64.3 ± 8.1	1-3	12week	③	Tango	Self-directed exercise	Falls
Morris [66]	2015	141	67.4 ± 10.4	67.9 ± 8.4	1-4	12month	①③④⑥	Resistance training	Life-skills information	Falls
Yu [67]	2015	71	63.5 ± 8.2	65.2 ± 7.4	3	3month	②④⑤⑥	Resistance training	Rehabilitation treatment	Not mentioned
Paul [68]	2014	40	68.1 ± 5.6	64.5 ± 7.4	NR	12week	④⑥	Resistance training	Low intensity sham	Acute bronchitis; Bone cancer; knee pain; acute back sprain
Nascimento [69]	2014	34	67.8 ± 6.8	66.3 ± 8.1	1-3	6month	⑨	Aerobic exercise	Standard therapy	Not mentioned
Volpe [70]	2014	34	68 ± 7	66 ± 8	2.5-3	8week	①②③④	Aquatic therapy	Land-based training	No
Choi [71]	2013	20	60.81 ± 7.6	65. 54 ± 6.8	1.8 ± 0.3	12week	①	Tai Chi	NR	Not mentioned
Amano [12]	2013	24	66 ± 11	66 ± 7	1.8 ± 0.3	16week	①⑤⑥	Tai Chi	NR	Not mentioned
Mckee [72]	2013	33	68.4 ± 7.5	74.4 ± 6.5	1-3	12week	①③④	Aerobic exercise	No intervention	Non-injurious falls
Corcos [73]	2013	48	59.0 ± 4.6	58.6 ± 5.6	2.5-3	24month	①③	Resistance training	MFCE	Wrist pain
Hass [74]	2012	18	64 ± 7	67 ± 8	1 - 3	10week	⑤	Resistance training	No intervention	No
Li [75]	2012	130	69 ± 8	69 ± 9	1 - 4	24week	①④⑤⑥	Resistance training	Stretching	Falls
Vivas [76]	2011	11	63.12 ± 13.61	64.23 ± 13.45	1 - 4	4week	①②④	Aquatic therapy	Land-based training	No
Reuter [77]	2011	90	/	/	2 - 3	24week	⑥	Nordic Walking	Relaxation Programme	Hypotension; falls; ankle twists; shoulder pain
Allen [78]	2010	48	66 ± 10	68 ± 7	NR	6month	③⑥	Resistance training	No intervention	Joint pain
Madeleine [79]	2008	26	64.9 ± 8.3	62.6 ± 1 0.2	1.5 - 3	3month	①②④	Tai Chi	Pharmacological treatment	Not mentioned

Note: ①UPDRS-Ⅲ; ②BBS; ③PDQ-39; ④TUGT; ⑤Step length; ⑥stride velocity; ⑦PDSS; ⑧PSQOI; ⑨MSQ; ⑩ ISI; ⑪UPDRS.

### Methodological quality assessment

The included studies underwent a methodological quality evaluation with the “Risk of Bias” tool as advocated by the ROB II [17]. The ROB was categorized as “low”, “some concern”, and “high” based on essential domains. The RevMan 5.4 software was used to produce aggregated bias risk diagrams and proportion charts. In cases of ambiguity within the evaluation process, a third-party opinion was sought for a balanced assessment.

### Statistical analysis

All data extracted from each study were analyzed using Stata 17.0 and Review Manager 5.4 software. The heterogeneity of the included studies was assessed with the I² statistic, a measure that quantifies the proportion of total variation across studies that is due to heterogeneity rather than chance. We used the weighted squares method with random-effects models in all cases [18]. Effect sizes were measured using the standardized mean difference (SMD), mean difference (MD), and 95% confidence intervals (CIs), providing an estimate of the magnitude and precision of the effects being studied. A sensitivity analysis was performed by removing the studies one by one. Publication bias was evaluated with Egger’s test.

## Results

### Literature search

**Fig 1:**
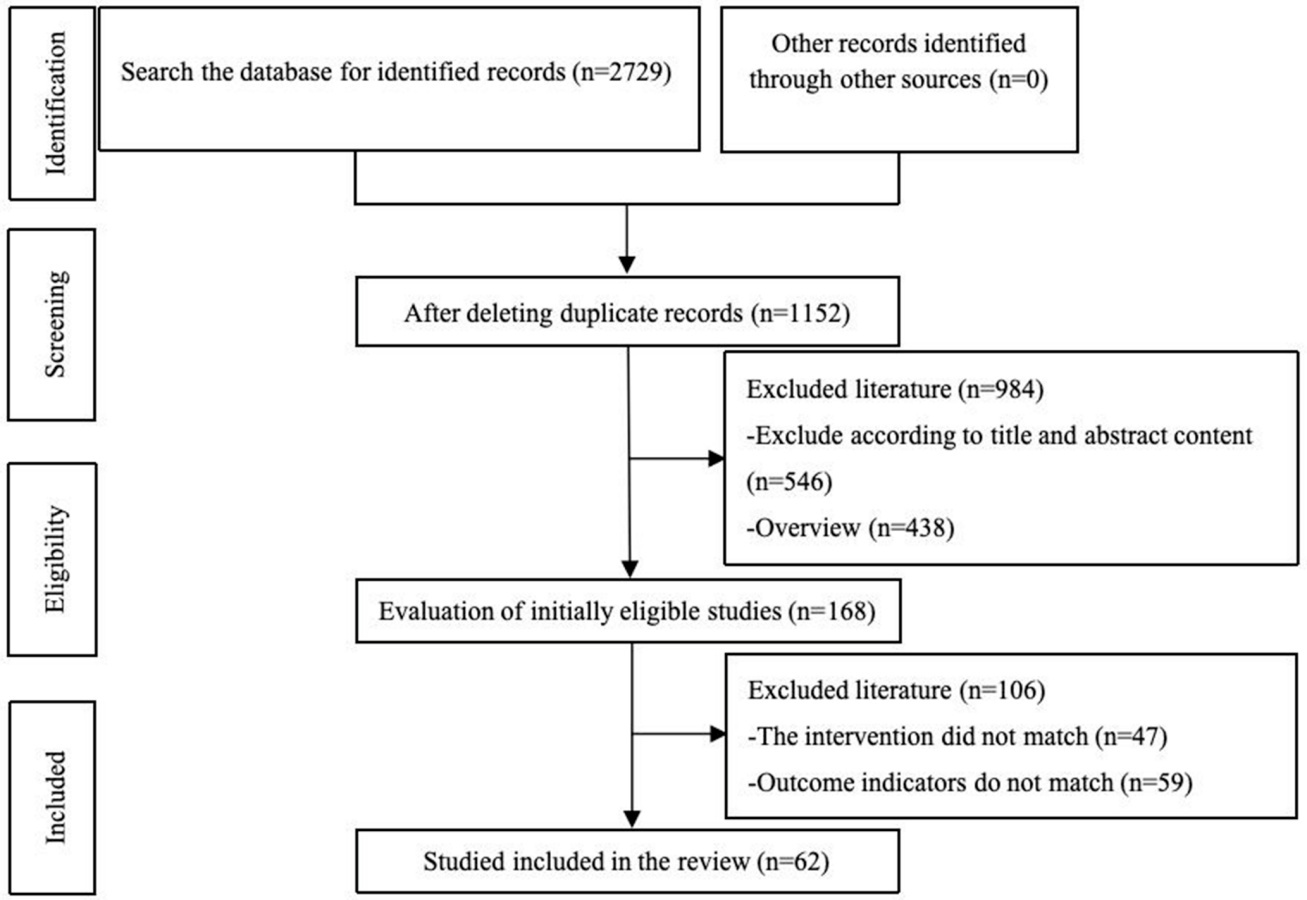
Flow diagram of literature screening.

A preliminary collection of 2,729 articles was obtained through keyword searches. After excluding 1,577 duplicate publications, a further 984 articles were excluded based on title and abstract screening, as well as the classification of literature types. An additional 106 articles were excluded after full-text review, resulting in 62 articles being included for the meta-analysis. The literature screening process is illustrated in Fig 1.

### Characteristics and quality assessment of included literature

**Fig 2:**
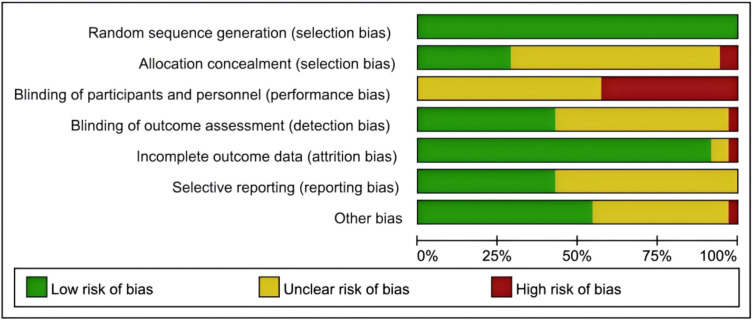
The risk of bias in the included studies.

The 62 included studies were all randomized controlled trials, spanning from 2008 ~ 2022, involving a total of 3,274 participants. The duration of the exercise interventions ranged from 3 ~ 52 weeks. The types of interventions included aerobic exercise, traditional Chinese exercise therapy, aquatic training, resistance training, tango, and yoga. The basic characteristics of the included literature are detailed in Table 1. The risk of bias in the included studies was evaluated according to the “Cochrane Handbook for Systematic Reviews of Interventions”, and the quality assessment of the included literature is illustrated in Fig 2, where green signifies low risk, red signifies high risk, and yellow indicates an uncertain risk.

### Meta-analysis results

#### Sleep quality.

**Fig 3:**
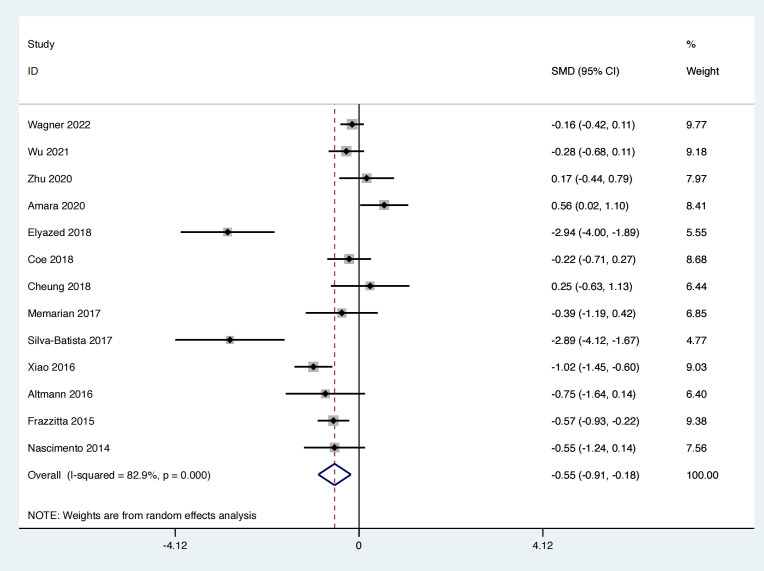
Forest plot comparing sleep quality between the exercise group and the control group.

A total of thirteen studies were included, involving 874 patients with PD, and sleep quality scores assessed. The heterogeneity test results indicated an I² = 82.9%, *p* < 0.001. The pooled effect analysis demonstrated that the exercise intervention group performed better than the control group, with a statistically significant difference between the groups [SMD = −0.55, 95% CI (−0.91, −0.18), *p* = 0.003]. The results showed that the total effect amount fell on the left side of the invalid line, and the exercise intervention was considered favorable, as shown in Fig 3. Subgroup analysis revealed that aerobic exercise [MD = −0.38, 95% CI (−0.54, −0.21), **p* *< 0.01], traditional Chinese exercises [MD = −0.63, 95% CI (−0.98, −0.28), *p* < 0.01] both exhibited superiority over the control group, with statistically significant subgroup differences (Q = 6.27, *p* < 0.05), as presented in Table 2.

**Table 2:** Subgroup analysis results of exercise intervention in Parkinson’s patients.

Subgroup-variable	Sample (k)	Outcome													
		Sleep quality		Motor capability		Balance ability		Gait performance						Quality of life	
				UPDRS-III		BBS		TUGT		Stride velocity		Step length		PDQ-39	
		95%CI	*p*	95%CI	*p*	95%CI	*p*	95%CI	*p*	95%CI	*p*	95%CI	*p*	95%CI	*p*
Type of exercises															
Aerobic exercise	16	−0.38 [−0.54, −0.21]	< 0.01	−0.49 [−0.79, −0.19]	< 0.01	/	/	/	/	0.48 [0.04,0.92]	0.04	/	/	/	/
Chinese traditional exercise	20	−0.63 [−0.98, −0.28]	< 0.01	−1.20 [−2.28, −0.12]	< 0.01	0.51 [0.21, 0.82]	< 0.01	−1.09 [−1.52, −0.65]	< 0.01	0.06 [0.02, 0.11]	0.03	2.75 [0.95, 4.56]	<0.01	1.55 [−5.41, 8.51]	0.44
Resistance training	17	−0.00 [−0.50, 0.49]	0.99	−0.74 [−1.12, −0.37]	< 0.01	0.28 [0.02, 0.55]	0.04	−0.35 [−0.64, −0.06]	0.02	0.13 [0.07, 0.19]	< 0.01	0.00 [−0.12, 0.13]	0.17	−0.61 [−1.13, −0.08]	0.02
Aquatic therapy	9	/	/	/	/	0.49 [0.23, 0.75]	< 0.01	−0.79 [−1.51, −0.08]	< 0.01	/	/	/	/	−6.35 [−12.17, −0.54]	0.03
Intervention duration															
≤ 12week	41	−0.79 [−1.42, −0.15]	0.02	−2.89 [−4.81, −0.97]	< 0.01	2.28 [1.29, 3.27]	< 0.01	−1.09 [−1.70, −0.49]	< 0.01	0.42 [0.09, 0.75]	< 0.01	0.42 [0.21, 0.63]	< 0.01	−0.29 [−0.87, 0.28]	0.31
> 12week	21	−0.33 [−0.76, 0.10]	0.14	−1.99 [−2.81, −1.16]	< 0.01	2.39 [0.68, 4.10]	< 0.01	−2.87 [−4.58, −1.16]	< 0.01	0.33 [0.01, 0.66]	0.04	0.22 [−0.04, 0.48]	0.10	−0.38 [−1.20, 0.44]	0.36
Weekly training frequency															
≤ 3	44	−0.53 [−0.95, −0.10]	0.02	−0.53 [−0.80, −0.25]	< 0.01	2.32 [0.76, 3.87]	< 0.01	−1.46 [−2.15, −0.77]	< 0.01	0.32 [0.01, 0.63]	0.04	0.12 [−0.25, 0.50]	0.52	0.47 [−0.90, −0.04]	0.03
> 3	18	−0.59 [−0.92, −0.26]	< 0.01	−0.21 [−0.34, −0.08]	< 0.01	2.57 [1.58, 3.55]	< 0.01	−1.17 [−1.97, −0.38]	< 0.01	0.45 [0.12, 0.79]	< 0.01	0.49 [0.26, 0.71]	< 0.01	0.04 [−0.23, 0.31]	0.77
Duration of a single training session															
< 60min	35	−0.68 [−1.20, −0.16]	0.01	−3.86 [−6.35, −1.37]	< 0.01	2.29 [1.29, 3.30]	< 0.01	−1.81 [−3.02, −0.60]	< 0.01	0.33 [0.11, 0.56]	< 0.01	0.44 [0.15, 0.73]	< 0.01	−0.41 [−0.99, 0.17]	0.16
≥ 60 min	27	−0.40 [−0.66, −0.15]	< 0.01	−3.13 [−4.99, −1.27]	< 0.01	2.45 [1.33, 3.58]	< 0.01	−0.47 [−0.77, −0.17]	< 0.01	0.45 [0.10, 0.79]	< 0.01	0.30 [0.10, 0.49]	< 0.01	−0.46 [−1.09, 0.17]	0.15

#### Motor capability.

**Fig 4:**
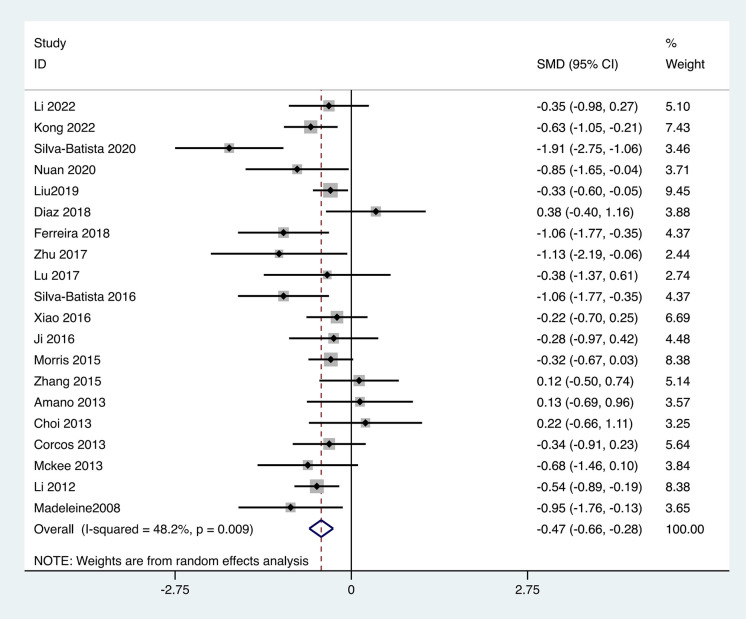
Forest plot for comparison of UPDRS-III score between the exercise group and the control group.

Twenty of the included studies utilized the UPDRS-III to assess the motor capabilities of PD patients, involving 1,074 patients. The heterogeneity test results indicated an I² = 48.2%，**p* *= 0.009. The pooled effect size demonstrated that the exercise intervention group performed better than the control group, with a statistically significant difference between groups [SMD = −0.47, 95% CI (−0.66, −0.28), **p* *< 0.01]. The results showed that the total effect amount fell on the left side of the invalid line, and the exercise intervention was considered favorable, as shown in Fig 4. Subgroup analysis results indicated that the aerobic exercise group [SMD = −0.49, 95% CI (−0.79, −0.19), *p* < 0.01], the traditional Chinese exercise group [SMD = −1.20, 95% CI (−2.28, −0.12), **p* *= 0.03] and resistance training group [MD = −0.74, 95% CI (−1.12, −0.37), *p* < 0.01] outperformed the control group, with statistically significant subgroup differences (Q = 14.87, *p* < 0.05), as shown in Table 2.

#### Balance ability.

**Fig 5:**
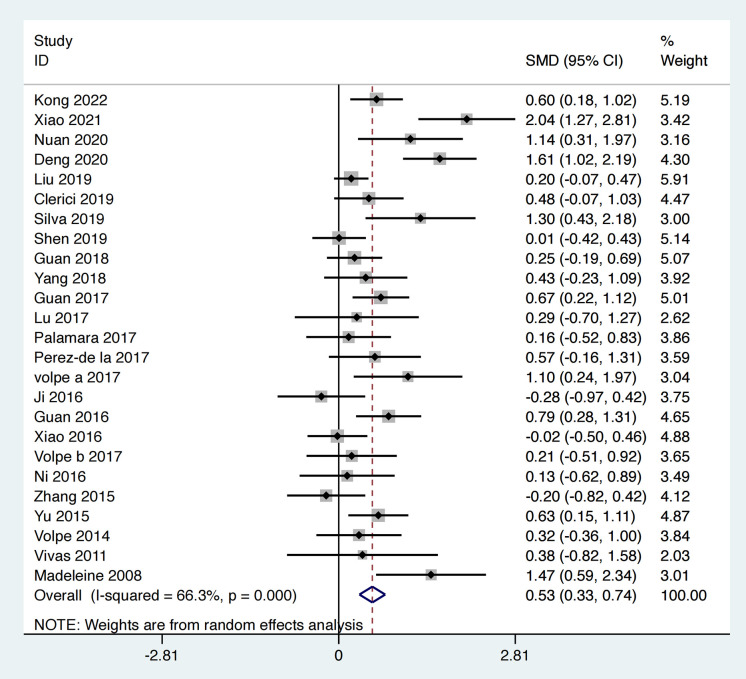
Forest plot for comparison of BBS score between the exercise group and the control group.

Twenty-five of the included studies utilized the BBS to assess the balance ability of PD patients, involving 1,287 patients. The heterogeneity test results indicated an I² = 66.3%, *p* < 0.001. The pooled effect size demonstrated that the exercise intervention group performed better than the control group, with a statistically significant difference between groups [SMD = 0.53, 95% CI (0.33, 0.74), **p* *< 0.0001]. The results showed that the total effect amount fell on the right side of the invalid line, and the exercise intervention was considered favorable, as depicted in Fig 5. Subgroup analysis results indicated that the aquatic therapy group [MD = 0.49, 95% CI (0.23, 0.75), *p* < 0.01], the traditional Chinese exercise group [SMD = 0.51, 95% CI (0.21, 0.82), *p* < 0.01], and the resistance training group [MD = 0.28, 95% CI (0.02, 0.55), *p* = 0.04] all outperformed the control group, with statistically significant subgroup differences (Q = 7.2, *p* < 0.05), as shown in Table 2.

#### Gait performance.

The included literature comprised thirty-four articles that investigated the impact of exercise on gait performance in patients with PD. The outcome measures were primarily focused on three aspects: TUGT, stride velocity, and step length.

**Fig 6:**
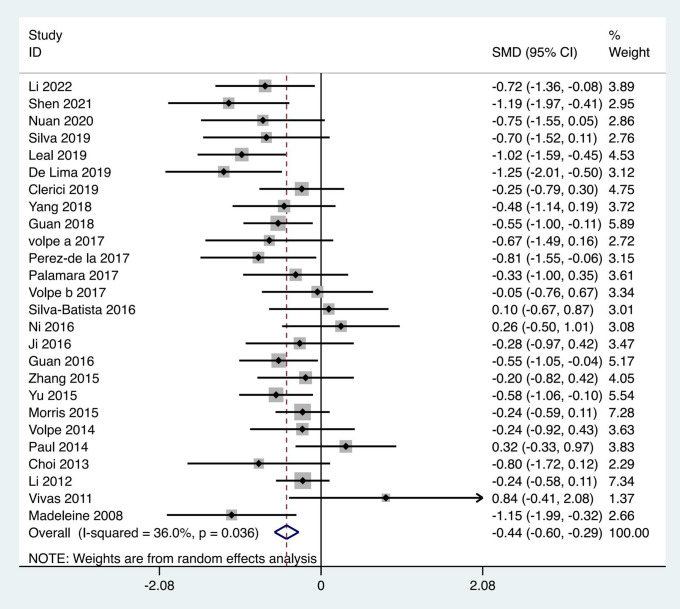
Forest plot for comparison of TUGT score between the exercise group and the control group.

TUGT: A total of twenty-six articles were included in the analysis, which utilized the TUGT to assess the gait performance of PD patients, involving 1,138 patients. The heterogeneity test results indicated an I² = 36%, **p* *= 0.036. The pooled effect size demonstrated that the exercise intervention group outperformed the control group, with a statistically significant difference between groups [SMD = −0.44, 95% CI (−0.60, −0.29), *p* = 0.0017]. The results showed that the total effect amount fell on the left side of the invalid line, and the exercise intervention was considered favorable, as shown in Fig 6. Subgroup analysis revealed that the aquatic therapy group [MD = −0.79, 95%CI (−1.51, −0.08), *p* < 0.01], the traditional Chinese exercises group [SMD = −1.09, 95%CI (−1.52, −0.65), *p* < 0.01], and the resistance training group [MD = −0.35, 95%CI (−0.64, −0.06), *p* = 0.02] all showed superiority over the control group, with statistically significant subgroup differences (Q = 9.98, *p* < 0.05), as detailed in Table 2.

**Fig 7:**
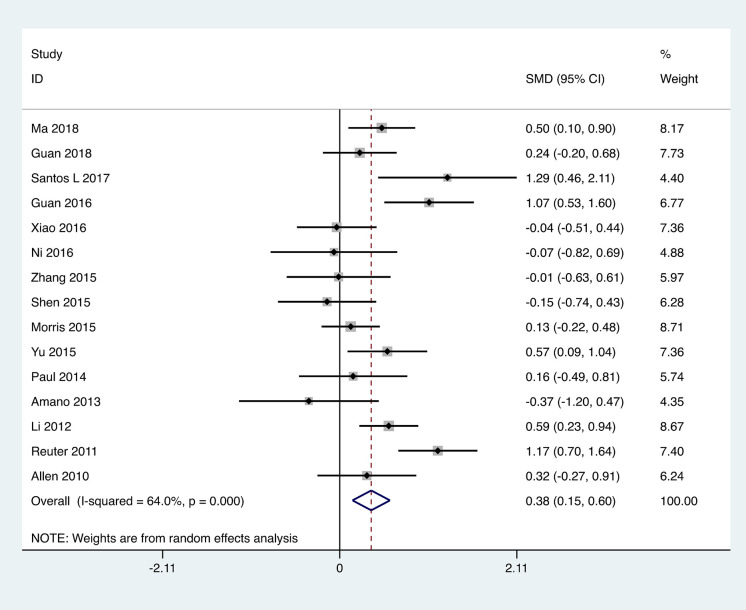
Forest plot for comparison of stride velocity score between the exercise group and the control group.

Stride velocity: A total of fifteen studies were included, employing stride velocity to assess the motor capabilities of patients with PD, involving 975 patients. The heterogeneity test of the studies indicated an I² = 64%, **p* *< 0.001. The combined effect size revealed that the exercise intervention group outperformed the control group, with a statistically significant difference between the groups [SMD = 0.38, 95% CI (0.15, 0.60), *p* = 0.001].The results showed that the total effect amount fell on the right side of the invalid line, and the exercise intervention was considered favorable, as depicted in Fig 7. Subgroup analysis indicated that the aerobic exercise group [MD = 0.48, 95% CI (0.04, 0.92), *p* = 0.04] and the traditional Chinese exercises group [SMD = 0.06, 95% CI (0.02, 0.11), *p* = 0.03] both demonstrated superiority over the control group, with statistically significant subgroup differences (Q = 6.47, *p < 0.05*), as presented in Table 2.

**Fig 8:**
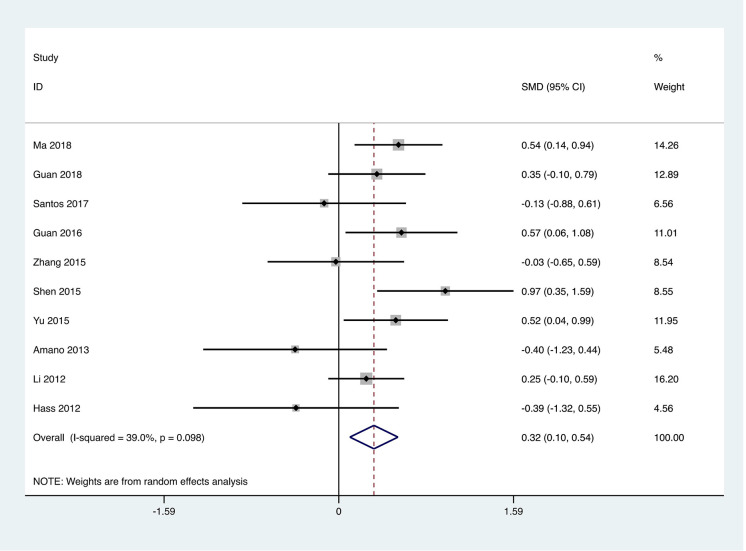
Forest plot for comparison of step length between the exercise group and the control group.

Step length: A total of ten studies were included, employing step length to evaluate the motor capabilities of patients with PD, involving 598 patients. The heterogeneity test of the studies indicated an I² = 39%, **p* *= 0.098. The pooled effect size demonstrated that the exercise intervention group performed better than the control group, with a statistically significant difference between the groups [SMD = 0.32, 95% CI (0.10, 0.54), *p* = 0.004]. The results showed that the total effect amount fell on the right side of the invalid line, and the exercise intervention was considered favorable, as shown in Fig 8. Subgroup analysis revealed that the traditional Chinese exercises group [SMD = 2.75, 95%CI (0.95, 4.56), *p* < 0.01] outperformed the control group, with statistically significant subgroup differences (Q = 5.92, **p* *< 0.05), as presented in Table 2.

#### Quality of life.

**Fig 9:**
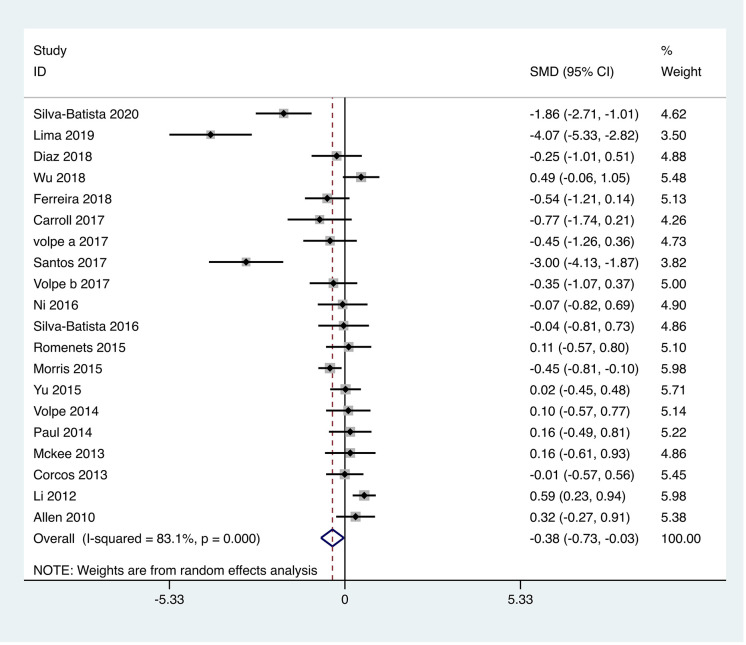
Forest plot for comparison of PDQ-39 between the exercise group and the control group.

A total of twenty studies were included, involving 891 patients with PD, with health-related quality of life scores assessed using the PDQ-39. The heterogeneity test results indicated an I^2 ^= 83.1%, *p* < 0.001. The pooled effect analysis demonstrated that the exercise intervention group outperformed the control group, with a statistically significant difference between the groups [SMD = −0.38, 95% CI (−0.73, −0.03), *p* = 0.04]. The results showed that the total effect amount fell on the left side of the invalid line, and the exercise intervention was considered favorable, as depicted in Fig 9. Subgroup analysis revealed that the aquatic therapy group [MD = −6.35, 95% CI (−12.17, −0.54), *p* = 0.03], and resistance training group [MD = −0.61, 95% CI (−1.13, −0.08), **p* *= 0.02] all exhibited superiority over the control group, with statistically significant subgroup differences (Q = 6.51, *p* < 0.05), as presented in Table 2.

### Sensitivity analysis

To assess the robustness of the pooled estimates, a leave-one-out sensitivity analysis was conducted by sequentially excluding each included study. Across all iterations, the effect sizes for all outcomes remained stable, with all corresponding p-values satisfying *p* < 0.05. Specifically, the SMDs and 95% CIs varied within narrow ranges: sleep quality (SMD: −0.38 [−0.69, −0.07] to −0.62 [−0.97, −0.27]; I2: 75% ~ 83%), UPDRS-III (SMD: −0.41 [−0.56, −0.25] to −0.49 [−0.66, −0.31]; I2: 41% ~ 48%), BBS (SMD: 0.46 [0.28, 0.65] to 0.55 [0.35, 0.75]; I2: 57% ~ 66%), TUGT (SMD: −0.40 [−0.55, −0.26] to −0.46 [−0.60, −0.32]; I2: 24% ~ 34%), stride velocity (SMD: 0.32 [0.11, 0.52] to 0.41 [0.19, 0.63]; I2: 53% ~ 67%), step length (SMD: 0.28 [0.04, 0.52] to 0.36 [0.16, 0.57]; I2: 21% ~ 44%), and PDQ-39 (SMD: −0.26 [−0.58, 0.06] to −0.44 [−0.79, −0.08]; I2: 79% ~ 84%). Critically, the exclusion of any single study did not alter the overall meta-analytic results. This consistency across sensitivity analyses confirms the reliability and robustness of the synthesized evidence.

### Publication bias

Egger’s test for publication bias was conducted for the outcome measures of motor function (UPDRS-III), gait performance (TUGT), and sleep quality in patients with PD. Compared with the funnel plot method, Egger’s test overcomes the limitations associated with the subjective assessments of funnel plots, yielding more objective results. The *p-values* for the UPDRS-III (*p* = 0.489), TUGT (*p* = 0.502), and sleep quality (*p* = 0.187) were all greater than 0.05, indicating the absence of publication bias.

## Discussion

This study employs a meta-analysis to quantify the effects of exercise therapy on PD, aiming to strengthen the existing evidence base and evaluate whether specific exercise interventions exhibit superior efficacy. This approach provides insights into potentially effective exercise treatment modalities for alleviating PD symptoms, which could inform future research. Our integrated analysis of multiple studies indicates that exercise therapy significantly improves PD patients’ sleep quality, motor function, balance, gait performance, and quality of life. Exercise is a comprehensive part of a healthy lifestyle and an essential strategy for both the prevention and treatment of PD.

Studies have indicated that physical exercise, particularly aerobic exercise, is crucial for improving the motor and non-motor symptoms of PD patients and for delaying the progression of the disease [80]. Aerobic exercise can enhance the motor control functions mediated by the cerebellum and prefrontal cortex. Aerobic exercise participates in the remodeling of the structure and function of D2-MSNs by modulating dopamine receptor activity and the downstream extracellular signal-regulated kinase signaling pathway, thereby preventing or improving PD-associated motor dysfunction [81,82]. Research has shown that aerobic exercise has become one of the primary non-pharmacological interventions for PD, beneficial for ameliorating PD-related symptoms and improving patients’ quality of life. The efficacy of aerobic exercise intervention involves the promotion of cerebral blood flow, brain arousal, activation of corticospinal excitability, and reduction of intracortical inhibition [83]. This study focuses on the same population and includes a variety of aerobic exercise modalities and outcome measures to provide a comprehensive evaluation of the effects of aerobic exercise intervention on PD patients. Engaging in 2 ~ 3 hours of physical exercise per week for 6 ~ 14 weeks, totaling 12 ~ 42 hours of training, has been shown to have a significant effect on improving PD symptoms [84]. Aerobic exercise increases the levels of brain-derived neurotrophic factor in the brain, promotes angiogenesis, and reduces neuroinflammation, thereby altering the structure and function of the brain in PD models to some extent [85]. The aerobic exercise studies included in this review had intervention durations ranging from 30 ~ 90 minutes per session, with frequencies of 2 ~ 6 times per week, over a period of 4 weeks ~ 6 months. The integrated analysis of the effects of aerobic exercise intervention in this study demonstrated that it significantly improved PD patients’ motor abilities, balance, gait performance, sleep quality, and quality of life, which is consistent with previous research findings [86,87]. Aerobic exercise enhances the balance of PD patients, reduces the likelihood of falls, alleviate fatigue and pain, and increases the frequency of daily activities [88].

Traditional Chinese exercise therapy, rooted in the health-preserving philosophy of Traditional Chinese Medicine, has a long-standing history. This study indicates that traditional Chinese exercise therapy has notable therapeutic effects on improving sleep quality and physical functioning in PD patients. For patients with PD, motor and walking abilities are crucial factors that directly affect their quality of life. Studies have shown that traditional Chinese exercise therapy can enhance motor and walking abilities by fine-tuning muscles and coordinating various body parts [89]. PD patients have a higher risk of falls, which can often lead to abrasions and lacerations, and in severe cases, fractures. Additionally, research suggests that among PD patients who have previously fallen, 60% experience fear of falling again and the resulting sense of shame, with 41% ~ 43% of them unwilling to engage in activities, leading to a decline in physical activity ability and further increasing their risk of falls [90]. Tai Chi has been shown to improve lower limb strength and muscle function in the elderly. It also enhances the coordination and control of lower limb and trunk muscles during walking [91]. The effect may be that during exercise, patients continuously shift their center of gravity and coordinate the movement of the limbs, thereby improving body flexibility and balance [92]. A study demonstrated that 24 weeks of Tai Chi significantly improved sleep quality in patients with chronic insomnia, as evidenced by a marked decrease in PSQI scores. This improvement was associated with a reduction in serum levels of TNF-α and TNF-β and an increase in sTNF-R1 and sTNF-R2. These findings suggest that Tai Chi may enhance sleep quality by regulating inflammatory factors. Intervention through traditional Chinese exercise therapy can alleviate the resting tremor frequency in early PD patients, improve balance, and enhance motor ability, thereby improving their quality of life [93].

This study demonstrates that resistance training, when analyzed in a meta-analysis of randomized controlled trials on motor ability, balance ability, and quality of life in PD patients, shows statistically significant differences, except for step length, which did not exhibit statistical significance. Resistance training is an effective method for increasing muscle mass and enhancing muscle strength by overcoming external resistance. It serves as a potent intervention for functional loss associated with aging and disease, capable of improving functional status and promoting neuroplasticity in the basal ganglia and cortical motor networks, which are crucial for gait performance. Resistance training stimulates skeletal muscle protein synthesis and promotes myocyte growth, which leads to an increase in muscle mass. It also enhances absolute muscle strength and muscle cross-sectional area through circulating anabolic hormones. Additionally, resistance training optimizes the neural innervation and activation of skeletal muscle, ultimately improving muscle strength in PD patients. This improvement contributes to better motor performance and quality of life [94,95]. Resistance training through activating the mammalian target of rapamycin pathway promotes muscle hypertrophy, thereby enhancing strength gains [96]. Lima et al. [97] through a meta-analysis, showed that resistance training has a positive effect on the prevention and treatment of PD; however, compared to this study, their research included fewer sources, shorter intervention times, and incomplete participant information. Gait disorders are common symptoms in PD patients and are considered important factors related to falls and increased morbidity. There is still some uncertainty regarding the impact of resistance training on gait performance in PD patients. Chung et al. [98] indicated that resistance training can enhance the balance ability of PD patients, but its effect on gait performance is not significant; Dibble et al. [99] showed that outcome variables and gait performance indicators improved after resistance training compared to the control group. To better understand the impact of resistance training on gait performance, this study analyzed three aspects: TUGT, walking speed, and step length, standardizing the measurement units for these three indicators to ensure reliable results. Among the three indicators for assessing gait performance, only step length showed no significant improvement, which may be due to differences in sample size and intervention durations. Overall, resistance training is effective as an intervention for PD. Nevertheless, these findings should be interpreted with caution.

Aquatic Therapy, conducted in a swimming pool, is influenced significantly by factors such as water depth, water temperature, the ambient temperature of the pool area, and the intensity of the exercises performed in water, all of which are crucial for patient safety [100,101]. According to the aquatic therapy guidelines by the Australian Physiotherapy Association, when aquatic therapy is used for patient rehabilitation, safety standards must be adhered to. Therefore, the included studies in this research have pools with a water depth ranging from 0.60 ~ 1.50 meters, a water temperature between 28°C and 38°C, and a room temperature between 25°C and 31°C [100]. Additionally, the presence of a physiotherapist during the study is essential for monitoring patient safety, the basic conditions of the swimming pool, and exercise intensity, with a physiotherapist present in all included studies. From the above, it is clear that the intervention for PD patients, based on the safety requirements of the water-based guidelines, is at an acceptable level of safety. Common disabling symptoms in PD patients include gait freezing and balance disorders, which not only affect the patient’s motor function but also lead to a high risk of falls and a low quality of life [100]. Therefore, improving these common disabling symptoms is very important. Currently, exercise therapy is one of the primary methods to improve motor function, and aquatic therapy, due to the unique physical properties of water such as density, viscosity, buoyancy, specific heat capacity, thermal conductivity, and latent heat [102], has distinct advantages in improving the motor function of PD patients [31]. In terms of balance ability and gait performance, this study shows that aquatic therapy can improve the balance ability and motor transfer ability in patients with mild to moderate PD. This may be related to the significant clinical effects of aquatic training on patients’ postural control and dynamic balance in a unique environment with reduced gravity and resistance [50]. Compared to land training, aquatic environments can buffer and protect patients; buoyancy reduces the load on joints, bones, and muscles, while resistance enhances muscular demands. Additionally, regarding the nervous system, water can elevate the activity of cortical sensory and motor areas, thereby promoting the integration of sensation and movement [103] and improving patients’ balance and gait performance. Although exercising in water may induce instability in patients’ bodies, it also encourages them to adjust their posture, achieve balance, and avoid falls [104]. Therefore, further studies are necessary to confirm these observations.

Subgroup analyses of exercise interventions in PD reveal that higher weekly training frequencies (>3 sessions) generally offer more significant improvements in sleep quality, motor capability, balance ability, and gait performance than lower frequencies (≤3 sessions), likely due to cumulative exercise effects enhancing muscle and neuromuscular adaptations. Similarly, longer single training sessions (≥60 minutes) often yield larger effect sizes than shorter sessions (<60 minutes), possibly because extended training time allows for more comprehensive muscle and motor skill development. Among exercise types, aerobic and traditional Chinese exercises show advantages in improving motor and balance abilities, while aquatic therapy benefits balance and gait. Intervention durations >12 weeks may provide more substantial motor and balance improvements than ≤12 weeks. Overall, the optimal exercise prescription depends on individual patient factors, and future research should focus on personalized exercise programs to enhance PD patients’ function and quality of life. Moreover, the optimal exercise prescription may vary based on individual patient characteristics and disease severity. Future research should focus on developing personalized exercise programs tailored to the specific needs and abilities of PD patients.

The present study has several limitations. The involved studies vary in terms of geographic region, sample size, assessment tools, and research methods, which contributes to a certain degree of heterogeneity. Although Egger’s test did not show any evidence of publication bias, some inevitable publication bias might exist. Small negative studies were less likely to be published, and gray literature, due to its diverse origins and unpublished nature, may be difficult to find. Since the sleep quality assessment tools in this study are mostly subjective, with only one study [29] using polysomnography for an objective assessment of sleep quality, this may introduce some clinical heterogeneity into the research results. Such heterogeneity could potentially obscure the true effects of specific types of exercise or patient subgroups, thereby increasing the difficulty of precisely estimating the overall impact of exercise interventions. Moreover, due to the limited availability of detailed data on intervention types, session durations, training frequencies, and patient demographics across the included studies, we were unable to conduct a meta-regression analysis to explore factors contributing to heterogeneity. In addition, different exercise interventions may have varying effects on the quality of life of PD patients. Direct comparisons between different exercise modalities were challenging due to the heterogeneity in intervention types and the lack of detailed data. Future research should include large-scale, high-quality RCTs to further substantiate and refine specific exercise intervention methods and should also focus on the individualization of exercise prescriptions based on patient characteristics.

## Conclusion

This meta-analysis suggests that exercise is a valuable non-pharmacological approach for improving sleep quality, motor function, balance, gait, and quality of life in individuals with PD, potentially complementing pharmacological treatments. Aerobic exercise (60~90 min, 2 ~ 3 sessions/week, 12 ~ 24 weeks) may improve motor function and sleep quality. Traditional Chinese exercises, such as Tai Chi or Qigong (60 min, 2 ~ 3 sessions/week, ≥ 24 weeks), may enhance balance and sleep, particularly in early-stage PD. Resistance training (moderate-to-high intensity, 2 ~ 3 sessions/week, 8 ~ 12 weeks) could improve motor capabilities. Aquatic therapy (45 ~ 60 min, 2 ~ 3 sessions/week in 28 ~ 38°C water) may support balance and gait. A cumulative exercise dose of 12 ~ 42 hours over 6 ~ 14 weeks, with >3 weekly sessions, is associated with notable benefits. Tailoring interventions to individual disease severity and physical capacity may support adherence and efficacy. Given the heterogeneity of included studies, these findings should be interpreted cautiously. Future large-scale randomized controlled trials are needed to validate these exercise protocols and explore long-term effects to optimize PD rehabilitation.

## Supporting information

S1 File PRISMA 2020 checklist. (DOCX)
